# Adolescent idiopathic scoliosis: natural history and long term treatment effects

**DOI:** 10.1186/1748-7161-1-2

**Published:** 2006-03-31

**Authors:** Marc A Asher, Douglas C Burton

**Affiliations:** 1 Department of Orthopedic Surgery, University of Kansas Medical Center, Kansas City, KS, USA

## Abstract

Adolescent idiopathic scoliosis is a lifetime, probably systemic condition of unknown cause, resulting in a spinal curve or curves of ten degrees or more in about 2.5% of most populations. However, in only about 0.25% does the curve progress to the point that treatment is warranted.

Untreated, adolescent idiopathic scoliosis does not increase mortality rate, even though on rare occasions it can progress to the >100° range and cause premature death. The rate of shortness of breath is not increased, although patients with 50° curves at maturity or 80° curves during adulthood are at increased risk of developing shortness of breath. Compared to non-scoliotic controls, most patients with untreated adolescent idiopathic scoliosis function at or near normal levels. They do have increased pain prevalence and may or may not have increased pain severity. Self-image is often decreased. Mental health is usually not affected. Social function, including marriage and childbearing may be affected, but only at the threshold of relatively larger curves.

Non-operative treatment consists of bracing for curves of 25° to 35° or 40° in patients with one to two years or more of growth remaining. Curve progression of ≥ 6° is 20 to 40% more likely with observation than with bracing. Operative treatment consists of instrumentation and arthrodesis to realign and stabilize the most affected portion of the spine. Lasting curve improvement of approximately 40% is usually achieved.

In the most completely studied series to date, at 20 to 28 years follow-up both braced and operated patients had similar, significant, and clinically meaningful reduced function and increased pain compared to non-scoliotic controls. However, their function and pain scores were much closer to normal than patient groups with other, more serious conditions.

Risks associated with treatment include temporary decrease in self-image in braced patients. Operated patients face the usual risks of major surgery, a 6 to 29% chance of requiring re-operation, and the remote possibility of developing a pain management problem.

Knowledge of adolescent idiopathic scoliosis natural history and long-term treatment effects is and will always remain somewhat incomplete. However, enough is know to provide patients and parents the information needed to make informed decisions about management options.

## Introduction

Scoliosis, simply defined as a lateral curvature of the spine, has been recognized clinically for centuries. The deformity is actually much more complex and to describe more completely and quantify scoliosis deformity, three planar and three dimensional terminology and measurements are required [1]. However, for practical purposes the deformity is most conventionally measured on standing coronal plane radiographs using the Cobb technique [2].

For a few of the patients an underlying cause can be determined, including congenital changes, secondary changes related to neuropathic or myopathic conditions, or later in life from degenerative spondylosis. However, the cause of most scoliosis is not known and since about 1922 such patients have been diagnosed as having idiopathic scoliosis [3].

Based on the observation of three distinct peak periods of onset, idiopathic scoliosis has been sub-divided into three groups; infantile, before age 3 years; juvenile, age 5 to eight years; and adolescent, age 10 years until the end of growth [4]. This classification is now widely used [5,6]. Eighty percent or more of idiopathic scoliosis is of the adolescent variety [7]. As it is often not possible to determine the age of onset, age at presentation/detection is more accurate [8]. Thus, it is likely that there is overlap at the age two/three years infantile/juvenile interface and at the age nine/ten year juvenile/adolescent interface. This is much less likely at the infantile/juvenile interface because most infantile curves present in the first six months of life, the most common curves are left thoracic apex, and males are more frequently affected, whereas the most common juvenile curves are right thoracic apex and females are more frequently affected [9]. This makes juvenile curve similar to adolescent curves. At the juvenile/adolescent interface it is almost certain that many of the younger adolescents had their curve well established during their later juvenile years. As the prognosis with juvenile presentation scoliosis is worse than it is for adolescent presentation scoliosis [5,6], inclusion of juvenile cases in adolescent series will tend to adversely affect the natural history of adolescent scoliosis.

The remainder of this presentation is devoted to adolescent idiopathic scoliosis, it being recognized that a few juvenile idiopathic scoliosis cases are undoubtedly included in the series cited.

Adolescent idiopathic scoliosis can probably best be considered as a complex genetic trait disorder. There is often a positive family history but the pattern of inherited susceptibility is not clear. Current information suggests that there is genetic heterogeneity [10]. This indicates that multiple potential factors are acting either dependently or independently in its pathogenesis [8].

The prevalence rate of adolescent idiopathic scoliosis, using a cut-off point of 10° Cobb or more, is approximately 2 % to 2.5% [11,12]. Prevalence as high as 9.2% has been reported: although only 0.23% required treatment [13]. The differences that have been found between specific populations are thought to be due to genetic factors [12]. However, it is possible that environmental factors may also be involved [14].

The prevalence is very dependent on curve size cut-off point, decreasing from 4.5% for curves of 6 degrees or more to only 0.29% for curves of 21° or more. It is also very dependent on sex, being equal for curves of 6–10° but 5.4 girls to 1 boy for curves of 21° or more [15].

The incidence, by year of birth, of treatment (brace or surgery) is remarkably stable averaging 0.26% (range, 0.14–0.43%) over a 23 year period from 1955 through 1977 [16]. The female to male ratio in this treated (brace or surgery) series was 7 to 1. Although the ratio of braced to operated patients wasn't provided, it is generally thought that approximately 0.1% will warrant surgery [17].

The purpose of this review is to summarize what is known about the natural history of adolescent idiopathic scoliosis after the growth years, as well as the long term effects of treatment. It is based on two untreated series from Sweden [5,18,19] and the mostly untreated series from Iowa [20-24]. Treated series cited had at least one and usually two or more of the following features: 10+ years follow up, 80+% follow up, controls, or health related quality of life questionnaire data. The end points considered are death, health impairment, deformity, and quality of life.

## Natural history

### Death

To the authors knowledge there are only two series of untreated idiopathic scoliosis patients with long term follow up; the first from Stockholm [19] and the second from Gothenburg [5,18].

In the Stockholm series ninety percent of 113 patients first seen from 1913 to 1918 were followed a minimum of 45 years, or until their death. Mortality was 2.2 times that of the normal population, and may have been higher if the eleven patients lost to follow-up could have been traced. The report had two weaknesses. First, the diagnosis was based on clinical notes which were usually supplemented by full length photographs; radiographs were available for only a few. Although paralytic patients were excluded, the series may have included a few patients with congenital scoliosis. Second, 9% of the series were age 7 to 9 at admission. The Gothenburg series included 130 patients with scoliosis of any cause enrolled from 1927 to 1936 at age 0 to 30 years. In their initial report results were largely given for the group as a whole [18]. The series was updated in 1989 when the minimum follow-up for living patients was 56 years [5]. One hundred fifteen (88%) of the patients were followed, 55 of whom had died. The effects of age of onset, defined as age of disease for polio patients, or first presentation for other patients; diagnosis (polio, rickets or unknown); and curve severity (<70° or >70°) on mortality were studied. There were no deaths in patients with adolescent (age 10–16 years) scoliosis of unknown etiology.

These findings are generally supported by those from the mostly untreated series of patients in Iowa, USA [20-24]. Of 444 idiopathic patients originally studied, 50 (11%) had been operated [21]. Of 358 patients whose deformity began after 8 years of age, 245 were located. Six were not eligible and 24 refused to participate, leaving 215 (60%) available for study. Their age averaged forty-two years (range, 32 – 64) and their follow-up 24 years (range, 20–36). At that point mortality was not significantly greater then expected, 7% versus 5.4% expected [20]. This cohort was next followed at an average of 39.3 years (range, 3151). Of 332 eligible patients, 219 (66%) could be traced and 33 had died for a mortality rate of 15%, not different than the 17% expected in a matched population [24]. Only one of the deaths, a 54 year-old with a 142° thoracic scoliosis, could probably be attributed to cor pulmonale secondary to scoliosis. This cohort was last studied at a mean age of 66 years and mean follow-up of 51 years. An additional 36 patients had died. The mortality rate could be determined for 203 (65%) of the 314 patients eligible for study. Assuming that half of the 127 patients not located were deceased, the probability of surviving to age 65 years for the study group was 0.55 and for a matched population 0.57. Scoliosis potentially contributed to death in 3 of the 36 deceased patients [22]. Their age, curve pattern, and curve size at death were 63 years, thoracic, 140°; 69 years, thoracic, 148°; and 53 years, double, 102°/70° and breast cancer.

Thus, it is safe to say that adolescent idiopathic scoliosis does not result in an increased mortality rate. However, it is also clear that it cannot be said that adolescent idiopathic scoliosis never causes death from cardiopulmonary failure. In a study of 800 patients with idiopathic scoliosis attending a chest clinic over 25 years eleven had died of cardiorespiratory failure due to scoliosis. In ten the curve had first been noticed before age 5 years, but in one it was first noted at 11 years of age [25]. The senior author (MA) has first hand experience with a patient diagnosed with idiopathic scoliosis at age 11 years, 5 months when her right thoracic curve was 40°. At age 12 years 1 month her curve had progressed to 50° and the recommended surgery refused. At age 44 years she died of cardiorespiratory failure and her curve at that time was 150° [26].

In a group of 45 patients with idiopathic scoliosis aged 16 to 67 years at enrollment the risk of developing respiratory failure and death was assessed over a 20 year period. Respiratory failure occurred only in patients with a predicted vital capacity of less that 45% and curve greater than 110° when enrolled into the study [27].

**Figure 1:**
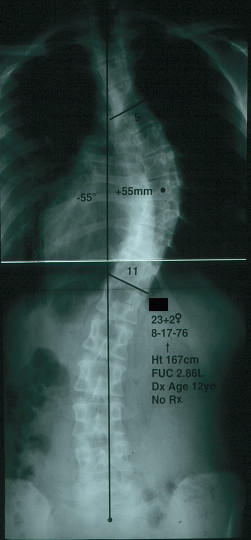
Posterior-anterior radiograph of a women with  right thoracic idiopathic scoliosis of 55 degrees at age 23 years. (Reprinted with permission from Asher M, Burton DC: **Natürlicher verlauf und langzeitauswirkungen der idiopathischen adoleszentenskoliose**. In *Wirbel Säulen Deformitäten: Konservatives Management*. Edited by Weiss HR. München: Pflaum; 2003:97-107.)

**Figure 2:**
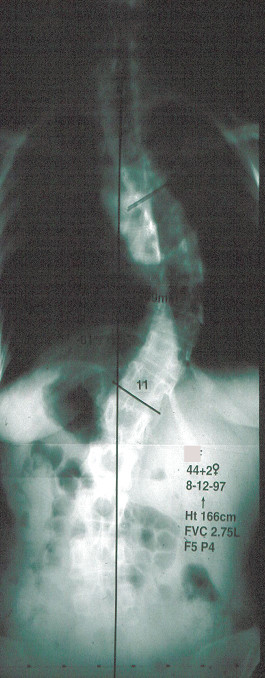
Posterior anterior radiographs of the same person as in Figure 1 at age 44 years, now with right thoracic idiopathic scoliosis of 61°. Her forced vital capacity (FVC) was 2.75 liters. Her function was normal and pain mild. (Reprinted with permission from Asher M, Burton DC: **Natürlicher verlauf und langzeitauswirkungen der idiopathischen adoleszentenskoliose. **In *Wirbel Säulen Deformitäten: Konservatives Management*. Edited by Weiss HR. München: Pflaum; 2003:97–107.)

Such information is reassuring for the adult patient who has adolescent onset idiopathic scoliosis in approximately the 50–70° range who is not concerned about their appearance and who is not bothered by pain. (Figure 1 and Figure 2)

### Natural history/health impairment

Pulmonary symptoms such as shortness of breath, not leading to premature death, may be associated with idiopathic scoliosis. These curves are usually larger, greater than 80° Cobb or with increased rotation, and usually single thoracic curves. Large double curves may also be associated with shortness of breath [24].

For patients with smaller curves there does not appear to be an increase in dyspnea. Evaluation at a minimum 20 years after completion of treatment showed no difference in dyspnea score for brace treated patients' with average Cobb angle of 40° when compared to age and sex matched controls [28]. Nevertheless pulmonary function does appear to be affected even in patients with relatively small curves [29]. To uncover this effect apparently requires stress testing [30,31]. It is very likely related to decreased chest wall motion [32].

Hypertension has been reported in one untreated scoliosis series to be higher than expected [5]. However, the series was not stratified by causation or age of onset for this analysis.

Neurological impairment associated with untreated idiopathic scoliosis would appear to be rare. Lumbar radiculopathy can occur and appears to be confined to the concave side of the curves, particularly the compensatory lumbosacral curve [33].

### Natural history/deformity

At an average of 40.5 years after skeletal maturity 68% of the 133 curves in 102 patients in the Iowa series progressed [23]. Curves initially 30°or less tended not to progress whereas curves more than 30° usually progressed. Single thoracic curves between 50° and 75° were the most likely to progress, an average of 29.4° or about 0.73°/year (29.4°/40.5 years). Others have noted that thoracic curves were the most likely to progress [34]. Additional risk factors for progression of single thoracic curves were those with apical vertebral rotation of more than 30 per-cent and Mehta-angle, a measure developed to differentiate resolving and progressing infantile idiopathic scoliosis [35], of more than 20° [23]. The lumbar components of double major curves were more likely to progress than the thoracic component. Right lumbar apex curves were twice as likely to progress as left apex lumbar curves. Lack of L5 deep seating and greater than 33% apex rotation were risk factors for progression [23].

### Natural history/quality of life

There are no series of completely untreated adolescent idiopathic scoliosis patients from which to learn the effect of the condition on the patient's quality of life.

The Iowa series, now with an average follow-up of 51 years, is the one with the longest follow-up [20-24]. However, it suffers from selection biases. Eleven per-cent (50/444) of the original cohort were excluded due to surgery and patients first presenting at age 8 and 9 years are included. In addition, the follow-up rate is low, with only 43% (117/271) of living, un-operated eligible patients providing health related quality of life information at latest follow-up [22]. However, it has been suggested that untreated patients who have either been lost to follow-up or who refuse to participate in natural history follow-up studies are those likely to have fewer symptoms [36].

The Ste-Justine series, with 1,476 (71%) of 2,092 patients followed at least 10 years after referral, is the largest [37-40]. However, the series also has several selection biases. Patients presenting at age 9 years were included. Thirty-eight per-cent [556/1467 (9 missing data points)] of the patients were operated, and "untreated" patients included those for whom bracing was recommended, whether or not it was performed. In addition, response rates were less than 70% for patients with curves less than 20° and for patients who had been under observation for two years or less [37].

Based on these series and many other individual attempts to gain insight in the natural history of adolescent idiopathic scoliosis, it is possible to gain a good idea, albeit incomplete, of the effect of adolescent idiopathic scoliosis on health related quality of life.

**Function, **based on outcome measures of work and level of disability, of patients with untreated adolescent idiopathic scoliosis do not appear different than controls [22]. However, this study was conducted over a period of time, and possibly in a population, when disability was less of an option. Based on responses to questionnaires scoliosis of even small size may be associated with difficulty in carrying out physical activities, particularly in females with curves greater than 40° [38].

**Back pain **prevalence is significantly higher than control populations. [22,39] However, back pain severity and duration may [39] or may not be increased [21,22].

Pain severity does not correlate with curve size [21,39]. Curve pattern may be associated with increased pain [23]. When related, thoracolumbar curves seem the most [24,34] and double curves the least [34] likely to be associated with increased pain. Arthritic changes are not associated with increased pain [22] whereas translatory shift in the thoracolumbar spine may be. [24].

**Self-image**, as measured by patient responses on a validated questionnaire scored from 1 (best) to 6 was significantly worse for scoliosis patients than controls, the comparison being 3.6 to 4.2, P = 0.001 [22]. For younger female patients with smaller curves self image was in some instance significantly higher than controls [38].

**Mental health **studies have yielded conflicting results. Based on a survey of the Iowa series, it was concluded that there were no mental health problems severe enough to require psychiatric treatment and that the deformities were better tolerated by middle age patients than teenagers [24]. At the most recent follow-up a validated depression questionnaire was used and there were no differences from a control population [22]. However, in an uncontrolled study, it was found that females with thoracic curves greater than 40° were particularly prone to psychological disturbance, it being present in 39% [34].

In the largely untreated Iowa series the untreated scoliosis did not appear to be detrimental to becoming married or childbearing [24]. Curve progression in untreated scoliosis patients does not appear to be influenced by pregnancy [41]. Older studies indicating that pregnancy and childbearing was affected detrimentally were the completely untreated series that included patients with mixed pathology and larger curves [19,42].

**Figure 3:**
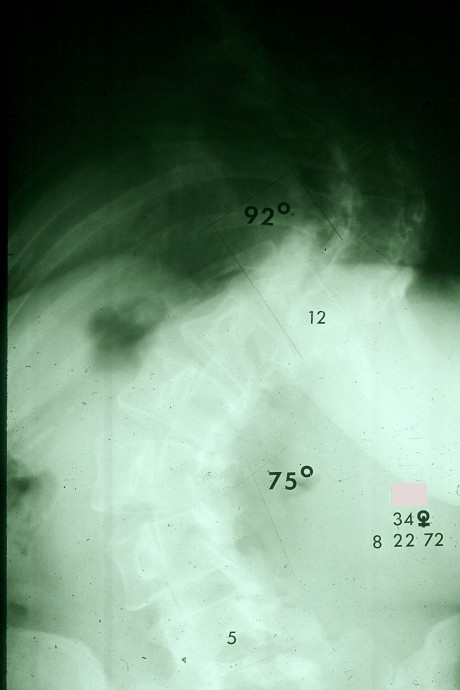
Posterior-anterior radiograph of a women with  double major scoliosis taken at age 34 years. (Reprinted with permission from Asher M, Burton DC: **Natürlicher verlauf und langzeitauswirkungen der idiopathischen adoleszentenskoliose**. In *Wirbel Säulen Deformitäten: Konservatives Management*. Edited by Weiss HR. München: Pflaum; 2003:97-107.)

**Figure 4:**
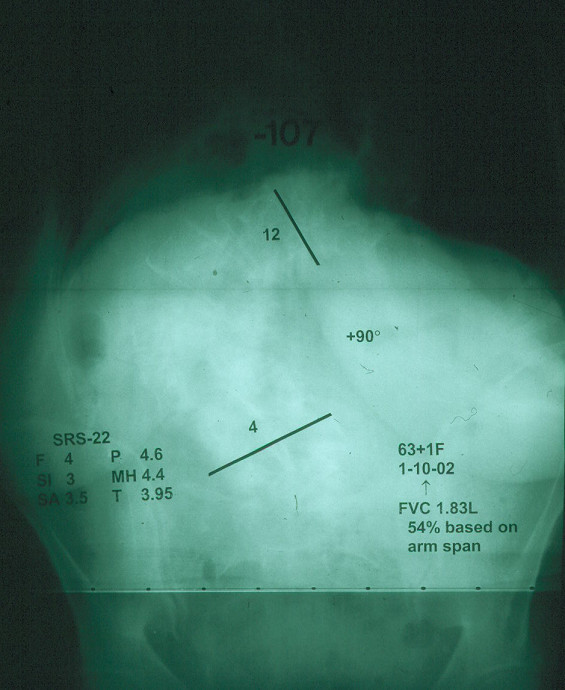
Posterior anterior radiographs of  the same person as in Figure 3 at age 63 years. Her forced vital capacity was 1.83 liters, 54% normal based on arm span. Her SRS-22 Health related quality of life scores ranged from 5 to 3, scale 5 best-1 lowest. (Reprinted with permission from Asher M, Burton DC: **Natürlicher verlauf und langzeitauswirkungen der idiopathischen adoleszentenskoliose. **In *Wirbel Säulen Deformitäten: Konservatives Management*. Edited by Weiss HR. München: Pflaum; 2003:97–107.)

Such knowledge of AIS natural history as is available, although admittedly incomplete, is detailed enough to provide reassurance about the long term effect of untreated adolescent idiopathic scoliosis for most patients (Figure 3 and Figure 4).

## Treatments

The currently accepted methods of treatment are bracing and surgery.

Bracing has been done for centuries. However, there does not appear to have been any documentation of beneficial effect until after the introduction of the Milwaukee brace [43] and later with the advent of thermoplastics and the thoraco-lumbo-sacral orthosis [44].

Although still somewhat controversial the best study to date indicates a small but significant beneficial effect of bracing [45]. In this prospective, multi-center, multi-national, center specific study 247 (86%) of 286 enrolled girls of skeletal age 10 – 16 years and single thoracic curves of 25° to 35° were followed until maturity or dropping from the study because of progression of at least 6° on two separate occasions. At 4 years follow-up the success rate was similar for observation and surface electrical stimulation at 36% and 33% respectively; whereas it was significantly better for bracing 74%, P <0.0001.

This finding was supported by a meta-analysis of 20 studies showing that the weighted mean proportion of success was 0.39 for lateral electrical surface stimulation, 0.49 for observation, and 0.60, 0.62, and 0.93 for bracing 8, 16, or 23 hours per day, respectively. The last was significantly more successful than any other treatment, P < 0.0001 [46].

What is still more controversial is whether or not a bracing program can decrease the frequency of surgery. However, promising results have recently been reported from two different centers using similar programs combining custom bracing and intensive inpatient rehabilitation. Compared to published series, the frequency of surgery was significantly reduced, by 50% or more [47,48].

Surgical treatment was initiated in 1914 [49]. When the results were evaluated in 1941 they were found to be poor [50]. As a result of the untiring work of John Moe, Paul Harrington, and many others these results had considerably improved by 1962 [51,52]. Due to advances in surgery the number of scoliosis curves greater than 100° had dropped considerably by 1973 [42].

The principle indication for surgery during adolescence is a thoracic curve that will reach 50° or more by skeletal maturity. The other curve patterns are more problematic because of the risk of low back pathology and pain after fusion into the low lumbar spine. However, thoracolumbar curves that will reach 50° to 60° at maturity may also be considered for surgery because of their association with a marked degree of deformity and vertebral translatory shift [24]. The indication for surgery, based on curve size, for double and lumbar curves cannot currently be stated with precision, but conservatism seems appropriate.

The indications for surgery as an adult are pain, appearance, and pulmonary problems, i.e. shortness of breath. However, it is unusual for these symptoms to be severe enough to warrant surgery. In the Iowa series eight patients had later surgery, and while if is difficult to exactly determine the size of the study population they came from, it would appear to be about 221, or 4% requiring surgery during adulthood [22].

### Risks

The possibility of increased risk of cancer as a result of the radiation from scoliosis x-rays has been raised. However, the x-ray technology used was much older and there were confounding disease variables [53].

Although there are some risks associated with surgery they have decreased substantially. Death is very unlikely but can occur, especially in patients operated as adults. [54] Neurological complications, for all cases of spine deformity reported by fellows of the Scoliosis Research Society, was 0.94% from 1965–71 [55]. By 2001–03 this had decreased to 0.49% for adolescent idiopathic scoliosis patients age 10–17 years [56]. Other complications include acute and delayed deep infection, pseudarthrosis, and implant prominence.

## Long term treatment effect

We are aware of only one long term series, with minimum 20 year follow-up, studying the effect of bracing. It is accompanied by a companion surgical outcome study [28,57-59]. There are at least three additional surgical series with a minimum 19 year follow-up [60-62]. And, there are five more series with a minimum follow-up of 10 years. [63-67] In contrast, there appears to be only two series of adolescent idiopathic scoliosis patients first treated surgically as adults [54,68]. Thus, the long term treatment effects are largely those of surgery, especially ones performed during adolescence. Some other focused studies help provide a picture of the long term effects of treatment, at least to about 20 years post treatment.

### Radiographic effects

With Harrington Instrumentation and arthrodesis curve correction is about 50% initially, with a wide range from 28 to 63% [57,60,61]. However, this decreases to about 40% at follow-up, although in one series it was only 15% [61]. Curve correction initially is similar for Cotrel-Dubousset instrumentation, but at follow-up is significantly better in the one series comparing the two, 42% compared to 15% [65].

Degenerative changes on lumbar spine radiographs have been noted with equal frequency in braced and Harrington instrumentation operated patients, 16% and 24%, both significantly greater than the control frequency of 0. Degenerative changes were not affected by the lower level of instrumentation [57]. However, when studied with flexion and extension dynamic radiographs, patients instrumented with Harrington instrumentation to L3 or L4 had significantly more translational motion than a comparison, asymptomatic group. Furthermore, increased translational motion correlated with increased back pain [69]. Utilizing both lateral flexion-extension dynamic radiographs and MRI, 60% of patients instrumented with Cotrel-Dubousset instrumentation had at least one degenerative abnormality at a minimum of 10 years post operatively. However, this is similar to those reported for asymptomatic populations of similar age [67].

The advisability of ending instrumentation above lumbar 4, or even 3, is still debated, with published studies apparently fairly evenly divided on the issue. However, if viewed from the vantage point of salvage surgery, 61% of 41 idiopathic scoliosis patients previously operated and requiring instrumentation and arthrodesis to the pelvis had lumbar 4 as their lower instrumented vertebra. Their primary surgery had been done an average of 19 (range, 2 – 45) years earlier [70]. It has been suggested that 10 years is not long enough to know the long term effects on the unfused lumbar spine [66]. Based on this current review, it may be that a minimum 20 years isn't long enough either.

The un-instrumented spine above the instrumentation is not mentioned in any of the long-term follow-up studies. Recently proximal junctional kyphosis has become a topic of increasing interest leading to the concern that it may be an effect of the newer, stiffer instrumentation constructs.

### Health impairment

At 12 to 21+ years follow up the re-operation rate for patients operated as adolescents ranges from 5.7% for curve related procedures only [57] to 22% [63] and 29% [60] for all indications. For patients operated as adults and followed relatively shorter periods, i.e. 2–17 years, re-operation rates appear to be 14–15% [54,68].

Pulmonary function is significantly improved at a minimum of 20 years follow-up in both braced and operated patients [28].

Pregnancy, childbearing and delivery experience of braced and operated patients are similar to controls, including the rate of low back pain, with only a few exceptions. Braced patients were older at first pregnancy and vacuum extractions were higher in the surgically treated group. Sexual function, largely due to appearance self-consciousness rather than pain, was significantly limited for both surgical and braced patients, more so for the surgical patients, compared to controls [58].

Spinal mobility is decreased, more so with Harrington than Cotrel-Dubousset instrumentation [61,65]. Trunk strength for both Harrington and Cotrel-Dubousset instrumented patients was similar to age and sex-adjusted reference values, although patients with Cotrel-Dubousset instrumentation performed significantly better in squatting [65]. These differences may be explained by the one vertebra longer instrumentation, lower end instrumented vertebral level, older age, and longer follow-up of the Harrington series. There were significantly more complications in the Cotrel-Dubousset series [65].

### Health related quality of life

Physical **function **is lower for braced and operated patients than controls but better when compared to other patient groups with more severe diseases processes [59]. The lower extent of the caudal fusion is associated with more lifting, running, standing and carrying problems [60].

**Pain **reported by operated scoliotic patients is more than non scoliotic controls [40,59,60,63]. In addition only those with surgery had pain management problems [40]. Pain level in post surgical patients has been associated with increased kyphosis and increased compensatory thoracolumbar/lumbar curves [63]. In patients operated in adulthood the pain appears to be less than in comparable, unoperated patients but is still greater than controls [68]. Patients with thoracic and double curves improved whereas thoracolumbar curves were not improved. Peak pain levels were similar to a non scoliotic control population but average pain intensity remained higher [54].

**Self image **is decreased during the treatment period for both braced and operated patients. Following completion of treatment brace patients return to normal. At an average of 7 years post-operative small differences persisted for the operated patients, the differences characterized as probably "more statistical than practical" [71]. However, in a series followed a minimum of 20 years, surgically treated patients significantly limited social activities due to their back [59].

**Mental health, **as determined by the mental health domain as well as the mental component summary of the SF-36 did not show any difference among surgical, brace and control groups at a minimum of 20 years post-surgery in one study [59].However, in another study of patients also operated with Harrington instrumentation the mental component summary score was significantly lower than age matched population norms, but the actual differences were small, 48.89 compared to 51.44 respectively, and likely not clinically meaningful [64].

## Conclusion

Knowledge of the natural history of adolescent idiopathic scoliosis has expanded greatly in the last two decades. It has become clear that only about one in ten curves progresses to the point that treatment with bracing is warranted, and only one in 25, or 0.1%, to the point that surgery is warranted.

Compared to controls untreated adolescent idiopathic scoliosis does not result in an increased mortality rate. However, it may on rare occasion progress to the point of causing death by cor pulmonale. The rate of dyspnea is slightly increased and is associated with thoracic curves of greater than 80°. Most patients with untreated adolescent idiopathic scoliosis function at or near normal levels, even though pain is more prevalent. Self image is often slightly diminished. Mental health is usually normal.

Bracing appears to prevent about 20% to 40% of appropriately braced curves from progressing 6° or more.

Surgery, consisting of instrumentation and arthrodesis has virtually eliminated large thoracic curves. Although most patients are satisfied with their results, follow-up at 20+ years shows significant, clinically relevant decrease in function and increase in pain compared to controls. Re-operation is required in 6 to 29%. And, a very few have pain management problems.

Even though the natural history and long term treatment effects on adolescent idiopathic scoliosis have become a lot clearer, there are still many unknowns. Non-operative treatment effectiveness is limited and needs to be improved. Selection of adolescent patients for surgery is usually straightforward for major thoracic curves, but is much more problematic for double, lumbar and even thoracolumbar curves. This is because of the low level of instrumentation and arthrodesis required, and the resulting stress concentration on the remaining mobile lumbar motion segments. While ten to twenty-five years is a long term follow-up after treatment, the patients are still relatively young, 30 to 40 years of age. Longer periods of follow-up are needed as they become increasing difficult to accomplish.
